# Effective Messages to Reduce Stigma among People Newly Diagnosed with HIV during Rapid ART Initiation

**DOI:** 10.3390/ijerph21091133

**Published:** 2024-08-27

**Authors:** Chadwick K. Campbell, Kimberly A. Koester, Xavier A. Erguera, Lissa Moran, Noelle LeTourneau, Janessa Broussard, Pierre-Cédric Crouch, Elizabeth Lynch, Christy Camp, Sandra Torres, John Schneider, Lyndon VanderZanden, Susa Coffey, Katerina A. Christopoulos

**Affiliations:** 1 Herbert Wertheim School of Public Health and Human Longevity, University of California San Diego, La Jolla, CA 92093, USA; 2 Department of Medicine, University of California San Francisco, San Francisco, CA 94143, USA; 3 Division of HIV, ID & Global Medicine, Zuckerberg San Francisco General Hospital, University of California San Francisco, San Francisco, CA 94110, USAkaterina.christopoulos@ucsf.edu (K.A.C.); 4 School of Nursing, University of California San Francisco, San Francisco, CA 94143, USA; 5 UCSF Alliance Health Project, San Francisco, CA 94102, USA; 6 Howard Brown Health Center, Chicago, IL 60637, USA

**Keywords:** RAPID ART, immediate ART, HIV stigma, HIV, care engagement, qualitative research

## Abstract

HIV stigma has a negative influence on antiretroviral therapy (ART) initiation and persistence and viral suppression. Immediate access to ART (RAPID ART) has been shown to accelerate viral suppression (VS) that is sustained up to one year after HIV diagnosis. Little is known about the role of RAPID ART in reducing individual-level stigma. We explored how stigma manifests in RAPID ART encounters and whether RAPID ART interventions influence individual-level HIV stigma during and in the time immediately after the diagnosis experience. We conducted in-depth interviews with 58 RAPID ART patients from three health clinics in San Francisco, CA, and Chicago, IL. Interviews were transcribed, coded, and thematically analyzed. In the results, we discuss three main themes. First, Pre-Diagnosis HIV Beliefs, which included three sub-themes: HIV is “gross” and only happens to other people; HIV (Mis)education; and People are “living long and strong” with HIV. Second, Positive and Reassuring Messages During the RAPID Experience, which included two sub-themes: Correcting Misinformation and Early Interactions with People Living with HIV. Third, The RAPID ART Process Can Disrupt Stigma. RAPID ART encounters served as a potent mechanism to disrupt internalized stigma by providing accurate information and dispelling unhelpful myths through verbal and nonverbal messages. Reducing internalized stigma and misinformation about HIV at this early stage has the potential to reduce the effect of HIV stigma on ART initiation and adherence over time.

## 1. Introduction

Substantial evidence suggests that immediate antiretroviral therapy (ART) leads to accelerated viral suppression (VS) sustained up to at least one year after diagnosis [1,2,3,4,5,6], which is critical for positive long-term clinical outcomes. Further, vs. reduces forward transmission of HIV, as those with an undetectable viral load cannot transmit HIV (U = U) [7]. Historically, patients who were diagnosed with HIV would attend multiple doctor appointments over weeks or months, during which counseling, preparatory lab work, and medical evaluations were conducted prior to being prescribed ART. In some cases, providers would wait until patients were “ready” to begin treatment [8]. However, over time, it became increasingly clear that starting patients on ART immediately or as soon as possible had short and long-term clinical benefits and reduced the likelihood of onward transmission [9,10]. Between 2017 and 2019, the World Health Organization, the International Antiviral Society-USA Panel, and the Department of Health and Human Services each began recommending immediate start of ART after HIV diagnosis [11,12,13]. Beginning in 2013, San Francisco’s Ward 86 was the first clinic in the United States to pilot the “RAPID” (Rapid ART Program Initiative for HIV Diagnoses) model, which aimed to provide immediate ART to patients, counseling, staging labs, and navigation of benefits [8]. While, ideally, the RAPID encounter occurs on the same day as diagnosis, such as when the testing and care programs are co-located, Rapid ART encounters can take place when individuals are diagnosed outside of a care site. In these cases, referral to care should happen within five days, and ART initiation should occur within one day of the first care appointment [5,14]. 

Understanding patients’ perspectives on the acceptability of this approach and their experiences and level of satisfaction with it is important to the successful implementation of RAPID ART. In qualitative studies, including our own, patients described wanting to start ART immediately to relieve their fears about untreated HIV, to stay healthy, and because they had positive experiences with and perceptions of the testing and/or care site [15]. Those who chose to delay initiation of ART did so because they wanted time to absorb their diagnosis, worried about side effects, or had concerns about costs (e.g., insurance coverage, co-pays) [15,16]. 

One well-documented and persistent barrier to HIV treatment and care is HIV stigma [17,18,19,20,21]. HIV stigma is associated with higher rates of infection and less care engagement in all phases of the treatment cascade among people living with HIV (PLWH) [22,23,24], and it negatively affects overall well-being and social relationships [25,26]. When some are diagnosed with HIV, they have already internalized negative ideas about HIV disease as well as PLWH and struggle with the realization that they are now a PLWH [25,27]. Coping with their new diagnosis and incorporating their new identity can be complicated, in part, by the commonplace misperceptions about HIV (e.g., HIV = death; HIV only happens to gay people; people who get HIV are promiscuous) [25,27,28,29,30,31,32]. The first author has previously described ‘(mis)education’ as an active process of sharing stigmatizing and inaccurate information by word of mouth that serves to reproduce HIV stigma [28]. As a result of these stigmatizing messages, despite decades of advancements in scientific knowledge, successes in HIV prevention research, and numerous educational and HIV stigma-reduction campaigns, some still think of HIV as a “death sentence” and express fears of death after receiving an HIV diagnosis [24,26,33,34,35]. Importantly, thinking of the possibility of death after an HIV diagnosis can be rational, given the history of the epidemic, and as a response to any life-threatening diagnosis; however, at this stage in the HIV epidemic, fear of death also reflects the silence around HIV and a lack of formal and informal education about HIV and the major scientific advances in the treatment and prevention of HIV in many communities in the US [28,36,37]. 

ART initiation is negatively associated with internalized stigma and poor self-image [23,38]. Thus, for Rapid ART interventions to be effective, it is necessary to understand and address the pre-existing stigmatizing beliefs about HIV, particularly among newly diagnosed persons from marginalized communities, such as Black sexual minority men (SMM), racial and ethnic minority cisgender women, and transgender women of color [39]. It is also crucial to note that stigma’s negative influence on ART initiation occurs over time. For example, Earnshaw et al. (2018) found that, in traditional or non-RAPID contexts, internalized stigma one month after diagnosis predicted avoidant coping three months after diagnosis, which, in turn, predicted lower ART initiation at six months [40]. Other studies have found that internalized and anticipated stigma are heightened immediately after diagnosis and subsequently lower over time [19,41,42]. These findings suggest that addressing individual-level stigma as soon as possible after diagnosis may be an effective way to increase earlier ART initiation. However, very little is known about how starting ART immediately affects HIV stigma, and little guidance is available about what messages may be productive for both addressing HIV stigma and supporting immediate ART initiation. Therefore, we sought to explore how individual-level stigma manifested in the RAPID ART encounter and whether the RAPID encounter influences or reduces stigma for the individual. 

## 2. Materials and Methods

We conducted semi-structured, in-depth interviews with a diverse sample of PLWH recruited from three urban care sites offering RAPID ART. We used a phenomenological approach and posed a key question to individuals who had gone through the RAPID ART process: what was that experience like? [43,44,45]. This approach was particularly useful as we aimed to gain an understanding of the common experiences across individuals recruited in different geographic locations, including San Francisco General Hospital’s Ward 86, an academic-center affiliated safety-net HIV clinic in San Francisco and where the RAPID model was developed; Howard Brown Health, a federally qualified health center in Chicago providing both testing and ongoing HIV care; and STRUT, a San Francisco AIDS Foundation sexual health and wellness center that offers HIV testing and linkage to care in San Francisco’s Castro neighborhood. 

Eligible participants had received an HIV diagnosis at least 90 days prior to the interview, had experienced immediate ART or were offered immediate ART but delayed starting, and were able to complete the interview in English or Spanish. Clinic staff reviewed medical records to identify potentially eligible patients and offered participation by phone or during regularly scheduled clinic visits. Two authors (K.K., X.A.E.) conducted interviews in person at Ward 86 from May 2018–January 2019 and at Howard Brown from April to August 2019. X.A.E. conducted three additional phone interviews with patients at Howard Brown from March to April 2020. Two interviewers (C.K.C., X.A.E.) interviewed STRUT clients via Zoom between August 2020 and May 2021 due to the COVID-19 pandemic. Interviews lasted an average of 60 min. 

Participants provided written informed consent and received $50 for their participation. All interviews were audio recorded and transcribed verbatim. Three members of the study team read the initial interviews and corresponding field notes and met regularly to discuss emergent themes to develop the codebook. The codebook included both a priori and inductive codes. Two members of the team independently coded six transcripts to ensure consistency and agreement regarding the code application. The study team discussed and resolved differences during team meetings. We further ensured reliability and consistency by maintaining decision trails. Once the codebook was finalized, all transcripts were coded in Dedoose [46] by a primary coder and a secondary coder. The current analysis includes text segments where the following nine codes were applied: pre-diagnosis HIV knowledge, pre-diagnosis HIV attitudes, HIV diagnosis narratives, ART uptake motivations, interactions with the RAPID team, stigma, diagnosis-related fears, reactions to same-day ART offer, and compelling messages. Thematic concepts related to stigma were noted and discussed during data collection, reflected upon during team meetings, and then elaborated through the comprehensive review and synthesis by C.K.C. of the commonalities and divergent narratives present in the aforementioned code reports [47].

The Institutional Review Board of the University of California San Francisco reviewed and approved this study.

## 3. Results

### 3.1. Participants

We interviewed a diverse sample of 58 participants (20 at Ward 86, 20 at Howard Brown, and 18 at SFAF). Participants were between 19 and 52 years old (Median age = 27); the majority were non-white (31.0% Black; 39.7% Latinx; 13.8% Multi-racial) and identified as cisgender men (77.6%). The majority (63.8%) identified as SMM. Over a third reported being unstably housed (31.0%) or unhoused (6.9%). The median time to start ART after diagnosis was six days, and the median time since participants were diagnosed with HIV was 356 days at the time of their interview. 

In the results that follow, we discuss participant narratives in three broad themes. First, we describe Pre-Diagnosis HIV Beliefs, which include three sub-themes: (1) HIV is “gross” and only happens to other people; (2) HIV (mis)education; and (3) PLWH are “living long and strong”. Second, participants described destigmatizing Positive and Reassuring Messages that they received during the RAPID process, which included two sub-themes: (1) Correcting misinformation and (2) Early interactions with PLWH. Lastly, we highlight that The RAPID ART Process Can Disrupt Stigma soon after diagnosis, a time when internalized and anticipated stigma and fear are heightened. Participant quotes are followed by their age, race, gender, sexual identity, and city [e.g., (40; Black; Cisgender SMM; Chicago)].

### 3.2. Pre-Diagnosis HIV Beliefs Shaped Perceptions of HIV Diagnosis

We asked participants to describe what they knew and thought about HIV before being diagnosed. Most participants described having limited or mostly negative understandings of HIV and people living with HIV. However, a minority of participants held less stigmatizing views. When these individuals were diagnosed with HIV, they were more hopeful about their future than those with disparaging views on HIV.

HIV is “Gross” and Happens to Other People: For some, HIV was associated with groups they did not belong to themselves, and therefore, they believed that HIV would not affect them. As one man described,

When it first started, a bunch of people were dying. I felt like it was a different world for me. It was a world that—and then it evolved to be—to seem like primarily for gay men. So, it really seemed like it was in a world that would never touch me. It would never—I mean, I never had met anybody with it. So, I didn’t have any friends around it. I learned everything I learned from school and TV, but it was never real to me. In actuality, it was kind of gross, too.

(37; Black; Cisgender Heterosexual Man; SF)

Another participant similarly described his understanding of HIV as:

Gross. Disgusting. Unacceptable. Life sentence. Like you get it, and you die. There’s no cure. Very bad. I have a really bad vision about HIV before then. I didn’t even have friends who were HIV-positive. That’s how bad it was. Now that it’s on me, it’s like—just sad, but it happened.

(33; API; Cisgender SMM; SF)

Other participants described how they previously thought about people who were diagnosed with HIV. One man described being from a very conservative religious home, where he struggled with his sexuality and learned little about HIV: “I always had the association of people who, um, contracted it… they were grimy, or they did something on purpose, or that led to that, you know what I mean?” (24; Black; Cisgender SMM; SF) One woman described growing up hearing people make fun of people who had HIV and feared that people would say, “she’s disgusting or she’s dirty.” (30; Black; Cisgender Heterosexual Woman; Chicago). Similarly, others thought that they were not at risk because they did not fit the negative characteristics of the people who they believed contracted HIV.

I thought I would never get it. I thought I would never get it. Like… I used to be so particular about who I chose to spend my evenings with, in a sense, you know? Like, I wasn’t like a lady of the night… Like, because I was like, you know what, I’m a pretty good person. And it’s like, I’m not a slut.

Interviewer: In your mind at that time, who did you think got infected with HIV?

Like… poor people. Like poor people. Or like, people who just were like… unaware. You know like… illiterate motherfuckers, pretty much.

(27; Multi-racial; Cisgender SMM; SF)

Another man wondered how he might have contracted HIV, “I’m not a wild person… I don’t know what did happen, but I’m trying to figure out how I got it. I’m thinking I got set up or it’s not for real.” (33; Black; Cisgender SMM; Chicago). For these participants and others, receiving an HIV diagnosis was a shock, in part because they had formed a belief that they were not the type of person who was at risk for HIV. 

HIV (Mis)education: In a number of cases, participants described having never received formal education about HIV and that they had only heard, through word of mouth, stigmatizing beliefs and inaccurate information about HIV. For example, one participant explained,

Oh god, no. No education about it… It was—it was really just a lot of stigma and a lot of jokes that people were throwing around and just very—a lot of ignorance on people’s part or like that were trying to scare me. And I think it was because, you know, we just don’t want to—uh, we just don’t want that in our lives. And so there’s gonna be a lot of negative jokes or negative comments made about it… So, no, I didn’t really know much about it.

(38; Latino; Gender non-binary; SF)

Another man described that, when he was growing up, he was told by a teacher and family members that “if you got diagnosed with HIV, you’re disgusting, and you’re trash.” (33; Native American; Cisgender SMM; SF). And one woman described her limited knowledge of HIV at the time of her diagnosis:

I didn’t know it could be treated. I just thought it was just going to turn into AIDS. So, that’s why I was really scared. I didn’t know a lot. I didn’t know that it could be undetectable. I actually really didn’t know nothing about it. I heard about it, but they really didn’t explain it in school or anything, the details. They just said, “HIV, AIDS; AIDS, you die.

(21; Black; Cisgender Heterosexual Woman; SF)

Importantly, not being educated about HIV or having incorrect information can contribute to a more upsetting diagnosis experience due to heightened fear related to morbidity and mortality [28,48,49]. For example, one man described:

Prior me knowing that I’m positive, like, you know…like, just those in full-blown AIDS, like, those dying or just those who’s about to die. I mean, same thing with me. Like, for me, my notion is, when you have the virus, it’s like you’re almost dying.

(37; Native American; Cisgender Bisexual Man; SF)

Due to a lack of accurate education, the belief that HIV equals death was expressed by several participants. One man described, “I didn’t understand anything about HIV… HIV meant AIDS; it meant that in two or three months you would die.” (49; Latino; Cisgender SMM; SF). Another thought that when you have HIV, “anything can kill me. A cold. Staying up too late. Or the worst, I’m going to hurt a lot of people, and death. Immediate death.” (36; Latino; Cisgender SMM; Chicago).

People are “Living Long and Strong” with HIV: A minority of participants described having less stigmatized views of HIV. Some had received education about HIV in school or from someone in their lives. Moreover, nearly all reported that they knew people living with HIV. One transgender woman described that when she was diagnosed, she was not afraid because she already knew that PLWH could have full, healthy lives.

I have friends, and they were taking their meds. And basically, they were telling me about the virals and being undetected and stuff like that. So, I kind of figured like, okay, if they could do it, I’m sure I could do it. And I see commercials and I’m hearing things and I’m like, okay, it’s stuff that’s really like… People are living with it every day and living long and strong lives.

(32; Black; Transgender Heterosexual Woman; Chicago)

Similarly, another participant described:

I have a lot of friends that are older. And they’ve been positive for many years and they’ve told me how it was before and how it is now and how like people are managing their HIV status. They’re not dying and all that, yeah. That’s kind of how like I realized that it wasn’t a big thing as it was anymore because I see how people are taking care of themselves.

(26; Hispanic White; Cisgender SMM; SF)

Other participants learned negative misinformation about HIV when they were young but subsequently sought out more accurate information about HIV in high school or college prior to their diagnosis. When asked what he learned about HIV when he was growing up, a participant explained:

Um, nothing. And that is why I chose to become a sex educator—is because we got, I think, really, next to nothing when I was in high school and in middle school. Um, uh, I think, like most folks, HIV and AIDS was like this joke and this death sentence that folks talked about. Um, so it was never like demystified, in any way, shape, or form, for me, as a kid, until I got into the work and got into learning for myself.

(25; Black; Cisgender SMM; SF)

These participants received their HIV diagnoses in the context of existing knowledge about HIV and personal connections to other people living with HIV. Understandably, some still described receiving their diagnoses as sad, emotional, or scary. However, in contrast to those with no personal connection to PLWH and less accurate information, these participants knew about treatment and were more hopeful about their future. 

### 3.3. Positive and Reassuring Messages during the RAPID Experience

Correcting Misinformation: You Will Be OK: During the RAPID ART process, participants described important messages they received from clinic staff that countered the stigmatized information they had come to believe. For some, this was in the form of accurate education about HIV and HIV treatment provided by caring and compassionate healthcare providers.

They really simplified it for me, which made it easy to understand. Yeah, so it was [their] enthusiasm that really [did it]—because it wasn’t nothing like really brainiac about it. It’s basically simple procedures. It’s really easy to do. Just stick to it. Take your medication and you’ll be fine.

(37; Black; Cisgender Heterosexual Man; Chicago)

This participant was quoted earlier; he thought that HIV was “gross” and that it existed in “a world that would never touch” him. Similarly, for a man who grew up in a conservative household, where non-heterosexual sexuality and HIV were topics that “a lot of people don’t think about”, the RAPID experience was where he learned about HIV.

I learned a lot. They gave me—I still have my little booklet that they gave me, because they gave you all this information, that HIV can lead into AIDS. I have all these little pamphlets of what it can be, and what can it transform into, and the whole “U = U”, how it works.

(20; Latino; Cisgender SMM; SF)

For some, these important messages were delivered in an especially caring manner by staff and providers. Another woman who worried about the stigma she would face from other people after her diagnosis and about hurting others described an impactful conversation she had with the nurse who informed her of her positive HIV test result.

He just pushed his laptop to the side and just held my hands and told me, “it’s not a death sentence”. Nobody has ever told me that. I never knew that because I only knew one person with it. Then, when he said you can start treatment today, and explained to me about the treatment, and how it can help you to become undetectable. Because, I was just afraid of hurting people. I didn’t know.

(30; Black; Cisgender Heterosexual Woman; Chicago)

Like others, she had concerns about death and did not have a contemporary understanding of HIV and HIV treatment. During her RAPID experience, she learned for the first time that HIV was not a death sentence and had a more positive outlook for her future as a result. Warm interactions like the one she described where counselors and providers made patients feel seen and safe were important to many participants. One woman described how her doctor demonstrated that HIV is not casually transmitted.

So I was eating a granola bar and she took a piece of the granola bar that I was eating and she ate it. And she was like, see, I ate behind you. And it was shocking because she’s a doctor. You know what I’m saying? So I was like, okay, she’s nice so I can’t get this, just by eating behind, I can’t get this. So that made me feel real comfortable after that.

(45; Multi-racial; Cisgender Heterosexual Woman; Chicago)

Another woman described how she felt when she received her diagnosis, even though she had not previously judged people with HIV:

When it’s on you, you’re like ‘I am like disgusting.’ … My body is like tainted forever. It felt like the worst thing.

(30; White; Cisgender Heterosexual Woman; San Francisco)

Her interaction with her test counselor had a large impact on her mindset. He was just an amazing like “it’s gonna be okay. This is where you’re at”. Like holding my hand and my partner’s hand through it all was like probably the only way I like felt ok, you know. He made me feel like “you’re okay. You are not a bad person. You’re gonna get through this”. Like, you know what I mean? And this is where you go. This is the steps.

Importantly, as these participants described, the messages were key to their ability to process their diagnosis and to begin coping with fewer stigmatizing beliefs about HIV. Further, the caring nature and behavior of providers were equally as important as the messages they delivered. For some, physical touch like handholding and sharing food to show patients that they don’t need to fear casual transmission comforted patients in a way that goes beyond simply conveying information. 

For some who did not have stigmatized beliefs about HIV, the comfort and information they received were still important. One man we discussed in the preceding section who had older friends living with HIV and learned from them that HIV was manageable went on to describe his reaction to receiving his diagnosis:

You just kind of get emotional about it, but then I got quickly I got over it because I got to Ward 86, yeah, because like they were like, “Well, it’s good that you got sent here and on the day you got diagnosed and all that. So, we just want to put you into treatment options”, and thank God, yeah.

(26; Hispanic White; Cisgender SMM; SF)

The RAPID experience provided these participants with HIV education, countered previously held stigmatized ideas about HIV for some of them, and let them know that they would be ok immediately after diagnosis, a time when internalized and anticipated stigma are heightened [40,42].

Early Interactions with PLWH: Some participants described that having a medical provider or counselor disclose their positive status was especially impactful. As one man described,

…the biggest thing that I’ll remember is the doctor, the doctor that was like, “You know, look at me. I’m here. I’m helping you. You could be someone like me”. He’s like, “You could later on help people that have the same thing happening to you, and then you’ll know how to navigate it”. … And so I think, you know, that talk sort of, like, calmed all the thoughts that I was having in my head.

(20; Latino; Cisgender SMM; SF)

Other participants described having similar experiences:

…making that decision [to start treatment] was easier—one of the nurse practitioners, and it was like, this is… he was like, “I take the same pill”. He’s like, “I literally take the exact same pill every morning. It’s totally fine”. He was like, “You don’t even notice you’re taking it”.

(28; Multi-racial; Gender non-binary; Queer; SF)

Another man, whose belief that he was going to die we discussed earlier, described the impact that the counselor’s disclosure had on him.

The HIV coordinator—he was like, “Oh, well. I had it when I was young”. I think he said like 14, 15. Then, he didn’t get started right away. I think he said he wished he did. I don’t remember. He was like, he’s still here. He’s still alive. He’s still going normal days.

Interviewer: How did that make you feel?

I’m not about to die.

(19; Black; Cisgender SMM; Chicago)

During the RAPID process, PLWH cared for and supported each of these men. Providers sharing their statuses helped to shift their understanding of HIV as a death sentence and showed them that it was possible to live a healthy life with HIV.

### 3.4. The RAPID ART Process Can Disrupt Stigma 

Before the RAPID ART approach, patients had to wait until their CD4 count reached a certain level before starting ART. Some providers have argued that it is too overwhelming for patients to discuss treatment at the time of diagnosis. They have advocated for the ‘patient readiness model’ [50], which aims to give patients time to adjust to their new diagnosis and start ART when they are “ready” to do so. Other providers believe that offering same-day ART gives patients control and empowers them. We previously reported that when participants in our study received accurate information about HIV and HIV treatment during the RAPID ART encounter, it relieved them of some of their fears of death and reduced anxiety [15]. In this analysis, participant responses highlighted that the timing of the offer of ART, immediately or very soon after diagnosis, provides a unique opportunity to disrupt stigma in ways that are helpful to patients. As we discussed earlier in these findings, some participants’ anxiety, sadness, and fear were, in part, based on misinformation and a lack of knowledge about HIV, such as believing that they were a casual risk to others or that HIV would quickly progress to AIDS. The RAPID model provides the opportunity to offer education, comfort, and treatment right away, preventing patients from walking away while still carrying those fears and misinformation.

Some participants described what they thought it would have been like if they had not been able to start treatment right away.

Honestly, [being offered ART right away] kind of like helps with like the anxiety of it all too, like know that like you can start doing something towards your health like immediately is helpful… if I had to wait around and like, you know, if I’m just sitting around the house waiting to be handed medication that I need, like that would not help me. That would not help my whole mindset of it. And like I would be worried about feeling—I would be worried about side effects. Like, you know, I’d be worried about like my health all the time. And something about, you know, getting that medication as soon as possible, it—it definitely helps—it helps me—help cope with it a lot better.

(Age Unknown; Black; Cisgender gay SMM; SF)

Similarly, when asked to imagine having to wait to start ART, another man described that it would be:

…basically, a month of just me staying in my room waiting and panicking internally. I wouldn’t want to go out. I wouldn’t want to really do anything because of just that mindset of anything might happen that I can get in contact with someone, and that was the main thing, was that anything that can happen, I might—something might happen. I don’t know… just that constant fear about I don’t want to give it to anyone else, and so until I’m better, I’m not doing anything.

(24; Latinx; cisgender gay man; SF)

Like others, he worried that just continuing to live his life for a short period while waiting to start treatment would be a risk to other people because “something might happen”. Lastly, as another man similarly described:

I think if I would have had to wait, I think I would have, one, been really scared… Um, I think them giving me the medication that day, one, it normalized the situation and just being like, you know, “this is something that happens. It’s happening to you. The next step is for this to happen. We can do that right now. Because if we do that right now, and you’re already on it, when you come back… you know, you’ll most likely be fine”… Um, not to say the scary part’s over, but it was just that little hump of like, oh, I don’t have to wait until I find a doctor.. Like, I’m not going to have to live with this for another month and be all weird.

(28; Black/Latino; Cisgender Queer SMM; SF)

For these participants, the timing of RAPID ART was key to their ability to get educated about HIV, walk away from their diagnosis with a treatment plan, and, in most cases, with medication, which served to mitigate self-stigma, thereby reducing anxiety, fear, and sadness.

## 4. Discussion

In this analysis focused on understanding the role of stigma in the RAPID encounter for newly diagnosed PLWH at three sites in San Francisco and Chicago, we found that RAPID ART encounters served as a potent mechanism to disrupt internalized stigma. This disruption occurred as a result of knowledgeable and caring professionals providing accurate information, dispelling unhelpful myths through verbal messages, and demonstrating through body language and demonstrative physical acts to compel patients to see that they were not “gross”. HIV stigma continues to be a barrier to care engagement and positive long-term clinical outcomes. Internalized and anticipated HIV stigma is associated with missed clinic visits, lower engagement in care, lower levels of ART adherence, and detectable viral load [51,52,53,54,55]. Importantly, previous findings suggest that higher levels of internalized HIV stigma one month after diagnosis predicted lower levels of ART initiation over time [40]. The immediate ART approach gives the opportunity not only to start HIV treatment immediately after HIV diagnosis but also to give HIV-related education, correct misperceptions, and address stigma. 

Participants in our study described several well-documented stigmatizing ideas and beliefs about HIV and people living with HIV that were critical in shaping their initial reaction to their diagnosis. For example, similar to the findings of other studies [15,27,28,56], having an understanding of HIV as a “death sentence” naturally contributed to fear and anxiety for participants. Further, participants described believing that HIV was something that only happened to other groups (e.g., gay men, people who are poor, “sluts”) with which they certainly did not self-identify or want to be associated. Participants described that they began to apply these previously held stigmatizing beliefs about HIV to themselves once they were diagnosed. This included the belief that HIV was “gross” and that HIV would simply “turn into AIDS” and lead to death. Coming to an HIV diagnosis with these beliefs makes it even more poignant and difficult for patients/people to hear that they have been diagnosed with an infection and one that is currently incurable. It is important to note that fear is a rational response to being diagnosed with HIV. However, here, fear is driven in large part by a lack of accurate information about HIV and stigmatizing narratives that are perpetuated in society [28].

In our study, participants described compelling messages they received during the RAPID encounter. These messages assisted participants in shifting from thinking of HIV as deadly, a danger to others in their lives, and something that can destroy their futures to an understanding of HIV as livable. Thus, we have intentionally used the term ‘people *living* with HIV’ instead of the increasingly used term ‘people with HIV’ to continue to emphasize this fact—that one doesn’t just *have* HIV, but that one can *live* with HIV. Further, being offered treatment right away gave patients hope, relieved anxiety and fear, and served to normalize HIV. 

In addition, physical expressions of comfort were important for participants. Previous studies suggest that patients newly diagnosed with HIV need providers to answer their questions and provide education, but for some patients, feeling seen as a person includes receiving physical expressions of comfort (e.g., hugs, handholding) [57,58]. In our study, physical touch not only served to comfort patients and make them feel seen, but these gestures also accompanied words like “it’s not a death sentence” and demonstrated that touching or sharing food are not risks to others. Given the ubiquitous nature of HIV stigma, patients receiving a diagnosis and treatment for HIV can perceive physical distance from providers as a form of stigma and discrimination [59,60]. So, while physical touch may not be a necessary part of RAPID encounter for a newly diagnosed patient with HIV, it can be an important way to convey care and to dispel some internalized stigma at the critical point in which a patient is beginning to process their diagnosis and consider treatment. Further, while several of our participants had providers and counselors who were living with HIV and willing to disclose their status during the RAPID encounter, this is unlikely to be true for the vast majority of people who receive an HIV diagnosis. However, given how impactful this experience was for these participants, it highlights the importance of early interactions with other PLWH, particularly for those who have internalized high levels of HIV stigma or may not have known anyone living with HIV. These interactions could be with other clinic staff, peers, or community volunteers who can serve to show participants that they can still live long, healthy, and productive lives.

The potent combination of lifesaving treatment alongside paradigm-shifting information at the time of HIV diagnosis can have a significant effect on stigma in part because internalized and anticipated stigma is particularly heightened at the time of diagnosis [19,41,42]. Thus, not only is immediate ART initiation associated with improved clinical outcomes [1,3,5,6], but the RAPID providers/interaction can provide additional HIV education [61], and is a critical opportunity to intervene on internalized stigma before it has the chance to do the work of producing fear, anxiety, and avoidant coping, and before it reduces the likelihood of ART initiation. Indeed, recent findings from Mbgako et al. [62] highlight the important role of immediate ART in reducing internalized stigma by potentially reducing HIV’s impact on their lives. It is important to note, however, that the potential stigma reduction during the RAPID process is limited to the individual level. The RAPID process is focused on positive individual health outcomes and, therefore, cannot directly address the stigma at a larger societal level. Thus, a newly diagnosed patient will still reenter a world of structural and interpersonal processes that continue to produce HIV-related stigma. Our findings suggest, though, that effective and compelling messages aimed at reducing stigma and promoting Rapid ART initiation can likely serve to improve individual-level HIV clinical outcomes by disrupting the process through which stigma reduces ART uptake over time.

There are limitations worth noting. We were unable to recruit patients who refused RAPID ART altogether and were therefore unable to explore the role of stigma in the diagnosis and RAPID ART process for those patients. Further, our samples were recruited in two large urban cities at three well-funded, LGBTQ-affirming, HIV services-providing institutions. Additional research is needed to explore stigma narratives and the impact of stigma on the RAPID process in other regions, rural and suburban settings, and different structural environments (e.g., with different healthcare funding, availability of culturally competent care, and public insurance availability). Despite these facts, ours is one of the first explorations of the emergence of stigma in the RAPID ART process and describes how RAPID ART presents an opportunity to address stigma immediately and in conjunction with ART initiation.

## 5. Conclusions

In summary, 40 years into the HIV epidemic, negative self-attitudes and beliefs related to HIV status continue to exert a powerful influence on newly diagnosed PLWH. However, our findings support the idea that reducing internalized stigma by correcting misinformation about HIV at the time of diagnosis and providing immediate ART has the potential to interrupt the deleterious effect of HIV stigma on ART care engagement for those newly diagnosed with HIV.

## Data Availability

The data by which these findings are supported can be requested from the corresponding author. The investigators will meet to review and respond to each request. The data are not publicly available due to concerns about participant privacy around some of the most sensitive domains of the interviews.
